# Strengthening Community Health Workers' Role in Noncommunicable Disease Prevention: Lessons From Tanzania for the Philippine Public Health System

**DOI:** 10.1002/hsr2.71216

**Published:** 2025-10-01

**Authors:** Richard Ian Mark T. Necosia, Joanne Vivien B. Necosia

**Affiliations:** ^1^ College of Public Administration & Governance Bukidnon State University Malaybalay City Bukidnon Philippines; ^2^ College of Arts and Sciences Bukidnon State University Malaybalay City Bukidnon Philippines

## Transparency Statement

The author affirms that this letter is an accurate and honest commentary grounded in the cited study and relevant Philippine public health concerns.

Dear Editor,

A recent study by Mashauri et al. [1] on the knowledge, attitudes, and role in noncommunicable disease (NCD) prevention and control among community health workers (CHWs) in northern Tanzania addresses a common problem globally: CHWs are underutilized despite their potential for delivering primary‐level interventions. Their results indicate that over half of Tanzanian CHWs are educated (92.1%) and have a positive attitude (100%) to prevent NCDs, but the actual practice is varied: 26.7% took part in NCDs screening, 41.4% took part in the community mobilization [1]. These deficiencies of training and access to screening instruments mirror some of the challenges facing many developing countries, such as the Philippines.

In 2024, the World Health Organization projected that Filipinos had a 24.5% chance of dying before age 70 years from cardiovascular diseases, cancers, diabetes, or chronic respiratory diseases, higher than the Western Pacific regional average of 15.6% [2]. Additionally, the mortality from NCDs rose from 651 per 100,000 population in 2000 to 714 per 100,000 in 2021, thus emphasizing the rising and immediate need for effective community‐based intervention [2]. Despite contributing to the provision of frontline healthcare services, the participation of barangay health workers (BHWs) in the organized prevention of NCDs remains low because of enduring system constraints. A recent qualitative study from the Philippines about an urban district indicated that although the BHWs were involved with NCDs‐ screening, patient support and health education, several challenges were noted for them, which included poor training, poor access to health facilities, health‐related economic burden, and low knowledge about preventive health behaviors in the community [3]. These barriers limit BHW participation in comprehensive NCD care and bring to the fore the imperatives for structural and capacity‐building policy responses.

We note that the Tanzanian study provides a road map. Occupational training, home visit frequency, task confidence, and screening tools availability were significant predictors of doorstep worker participation [1]. These results are similar to those reported in the Southeast Asian studies where a community‐based task shifting in NCDs control significantly enhance early detection and saves the long‐term cost when properly supported [4].

Hence, we present the following policy perspectives tailored to the Philippine context:

Embed BHW Responsibilities in NCD Programs. The Philippine Department of Health (DOH) and Local Government Units (LGUs) need to require the participation of BHWs in NCD‐intensive trainings, capacity development activities, including hypertension clubs, diabetes screening days, and regular monitoring. This official title not only defines roles and responsibilities but also legitimizes BHWs in the eyes of their communities.

Develop Modular NCD Training and Certification Pathways. BHWs should undergo standardized capacity‐building programs co‐developed with public health training institutions. The Philippine Package of Essential Non‐Communicable Diseases Interventions (PhilPEN) framework already provides content, but implementation at scale remains poor, often due to the lack of financial, human, and material resources [5].

Equip Barangay Health Stations with Diagnostic Tools. As in Tanzania, the absence of basic tools such as blood pressure monitors, glucometers, and educational materials diminishes CHW participation. Providing these essentials, along with visual aids and mobile health apps, can help standardize care delivery.

Incentivize BHW Engagement Through Support and Supervision. The Tanzanian study found that CHW confidence levels strongly influenced participation in NCD‐related tasks. In the Philippine context, regular supervisory visits, continuing education, and performance‐based incentives (e.g., stipends, recognition programs) could encourage sustained engagement and reduce burnout.

Foster Community Trust in BHWs as NCD Advocates. Over 50% of Tanzanian CHWs cited poor public perception as a barrier. A similar issue exists locally, especially when BHWs are tasked with activities outside their traditional maternal and child health roles. Community information campaigns and closer linkage between BHWs and rural health units can help improve legitimacy and uptake of NCD services.

Beyond these, the Philippine government must recognize that scaling NCD prevention requires not only hospital infrastructure but also human capital at the community level. As Mashauri et al. [1] rightly argue, CHWs' proximity to households allows for timely education, early detection, and routine monitoring all at minimal cost to the healthcare system. If supported adequately, BHWs can bridge the last‐mile gap in NCD care, particularly in geographically isolated and disadvantaged areas [3].

Ultimately, task‐shifting to BHWs is not just a stopgap for workforce shortages; it is a strategic investment in community health resilience. The Tanzanian experience underscores that technical capacity, community trust, and institutional support must go hand in hand. The Philippines would do well to heed these lessons.

## Author Contributions

**Richard Ian Mark T. Necosia:** conceptualization, supervision, writing – original draft. **Joanne Vivien B. Necosia:** investigation, writing – review and editing.

## Conflicts of Interest

The authors declare no conflicts of interest.

## Data Availability

The authors have nothing to report.
